# Disassembly of the self-assembled, double-ring structure of proteasome α7 homo-tetradecamer by α6

**DOI:** 10.1038/srep18167

**Published:** 2015-12-14

**Authors:** Kentaro Ishii, Masanori Noda, Hirokazu Yagi, Ratsupa Thammaporn, Supaporn Seetaha, Tadashi Satoh, Koichi Kato, Susumu Uchiyama

**Affiliations:** 1 Okazaki Institute for Integrative Bioscience, National Institutes of Natural Sciences, 5-1 Higashiyama, Myodaiji, Okazaki, Aichi 444-8787, Japan; 2 Department of Biotechnology, Graduate School of Engineering, Osaka University, 2-1 Yamadaoka, Suita, Osaka 565-0871, Japan; 3 Graduate School of Pharmaceutical Sciences, Nagoya City University, 3-1 Tanabe-dori, Mizuho-ku, Nagoya, Aichi 467-8603, Japan; 4 Faculty of Science, Kasetsart University, Bangkean, Bangkok 10900, Thailand; 5 Institute for Molecular Science, National Institutes of Natural Sciences, 5-1 Higashiyama, Myodaiji, Okazaki, Aichi 444-8787, Japan; 6 JST, PRESTO, 3-1 Tanabe-dori, Mizuho-ku, Nagoya, Aichi 467-8603, Japan

## Abstract

The 20S core particle of the eukaryotic proteasome is composed of two α- and two β-rings, each of which is a hetero-heptamer composed of seven homologous but distinct subunits. Although formation of the eukaryotic proteasome is a highly ordered process assisted by assembly chaperones, α7, an α-ring component, has the unique property of self-assembling into a homo-tetradecamer. We used biophysical methods to characterize the oligomeric states of this proteasome subunit and its interaction with α6, which makes direct contacts with α7 in the proteasome α-ring. We determined a crystal structure of the α7 tetradecamer, which has a double-ring structure. Sedimentation velocity analytical ultracentrifugation and mass spectrometric analysis under non-denaturing conditions revealed that α7 exclusively exists as homo-tetradecamer in solution and that its double-ring structure is disassembled upon the addition of α6, resulting in a 1:7 hetero-octameric α6–α7 complex. Our findings suggest that proteasome formation involves the disassembly of non-native oligomers, which are assembly intermediates.

The proteasome, a huge protein complex responsible for ubiquitin-dependent proteolysis in eukaryotic cells^1^,^2^, is composed of a 20S core particle, which serves as a proteolytic chamber, and one or two regulatory particles^3^. The core particle consists of two α- and two β-rings, which form a cylindrical, four-layered αββα structure^4^,^5^. The α-ring is composed of seven homologous but distinct subunits, α1–7, that are arranged into a hetero-heptameric structure in the order α1-α2-α3-α4-α5-α6-α7 with assistance of assembly chaperones^6^,^7^. Subsequently, seven homologous and distinct β subunits, i.e., β1–7, are recruited to specific positions on the α-ring^8^.

Among human proteasome α subunits, α7 exhibits a unique feature of *in vitro* self-assembly into a homo-tetradecamer with a double ring structure^9^,^10^. This raises the question whether the α7 homo-tetradecamer is an off-pathway, dead-end product during proteasome formation. Another possibility is that certain mechanisms exist for disassembling the homo-oligomer of the α7 subunit, resulting in its monomeric form, which is a component of the hetero-heptameric α-ring. To resolve this issue, a conventional experimental method is required for characterizing the oligomeric states of proteasome subunits.

Mass spectrometry (MS) under non-denaturing conditions and sedimentation velocity analytical ultracentrifugation (SV-AUC) offer a powerful combination for analyzing biomacromolecular complexes^11^. The MS method enables us to determine the stoichiometry of the complex by observing accurate biomolecular masses. However, it is rather difficult to obtain quantitative information, for example, regarding the amounts of the molecules in the complexed and free states in solution. In contrast, SV-AUC enables us to determine the quantitative distribution of free and oligomeric species existing in solution, although the accuracy of mass determination in this method is lower than that in the MS method. In addition, recent sophisticated SV-AUC analyses have provided us with information regarding the structures of proteins and their complexes in solution, based on comparisons of experimentally estimated hydrodynamic parameters, e.g., sedimentation coefficient and diffusion constant, with those computed from their three-dimensional structure models^12^.

Here we determine a crystal structure of the human α7 homo-tetradecamer and apply the complimentary MS and SV-AUC methods to investigate the oligomeric states of the proteasome α subunits, focusing on α7 and α6, its neighbor in the correctly arranged α-ring.

## Results & Discussion

### Structure determination of the α7 homo-tetradecamer

**Figure 1:**
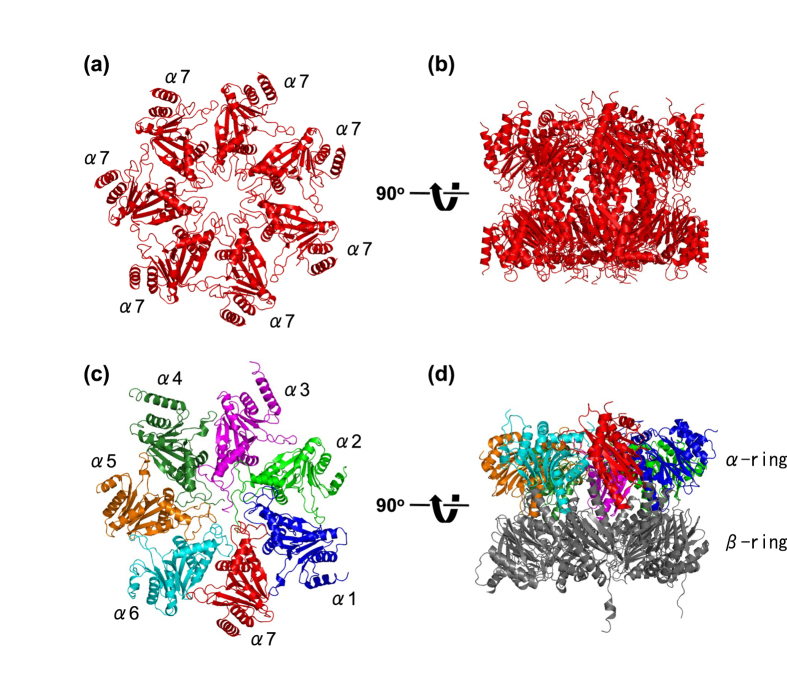
Crystal structure of the human α7 homo-tetradecamer. Ribbon models of the single and double α7 rings derived from the human α7 tetradecamer are shown in (**a**,**b**), respectively. Ribbon models of the α1–7 ring and the α1–7-β1–7 ring (half-proteasome) derived from the human 20S proteasome (PDB code 4R3O) are shown in (**c,d**), respectively. The left and right structures are related by a rotation of 90° around a horizontal axis. The α subunits are colored as follows: α1 (blue), α2 (green), α3 (magenta), α4 (forest green), α5 (orange), α6 (cyan), and α7 (red).

First, we determined the crystal structure of the human α7 tetradecamer at 3.75-Å resolution. The α7 tetradecamer exhibited an hourglass double-ring shape (Fig. 1a,b), which is consistent with the previously reported negative staining electron micrographs^9^ and small-angle neutron scattering (SANS) data^10^. The two α rings interact with each other mainly through two α helices that are involved in β-subunit interaction in the 20S proteasome^5^,^13^. The conformations of the individual protomers and their quaternary arrangement in each ring of the human α7 tetradecamer are essentially identical with those observed in the homo-heptameric α-ring in archaeal proteasomes^14^ and the hetero-heptameric α-ring in mammalian proteasomes (Fig. 1c,d)^5^,^13^.

### Oligomeric states of proteasome α7 and α6 subunits

**Figure 2:**
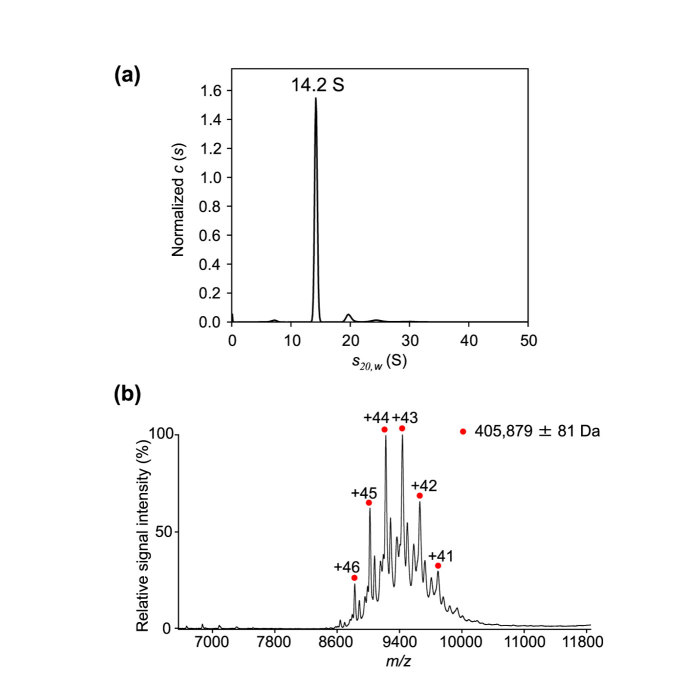
Characterization of the oligomeric state of the α7 subunit. (**a**) Distribution of α7 sedimentation coefficients derived from SV-AUC experiments. The 14.2 S peak corresponds to the α7 homo-tetradecamer. (**b**) Mass spectrum of α7 under non-denaturing conditions. Red circles show the ion series of the α7 homo-tetradecamer.

To characterize the oligomeric state of the α7 subunits in solution, we performed MS and SV-AUC analyses. The SV-AUC data showed that the α7 subunit exclusively exists as a single species with a sedimentation coefficient of 14.2 S (Fig. 2a), which is in excellent agreement with that estimated from the crystal structure (14.4 S), confirming the double-ring structure of this complex in solution. The mass spectrum of α7 under non-denaturing conditions exhibited ion series indicating a complex with a molecular mass of 405,879 ± 81 Da (Fig. 2b). This confirms the homo-tetradecameric structure of this subunit (with a calculated mass of 28,284 Da, based on its amino acid sequence). Therefore, these results are consistent with the present crystal structure and the previously reported electron microscopy and solution scattering observations^9^,^10^.

**Figure 3:**
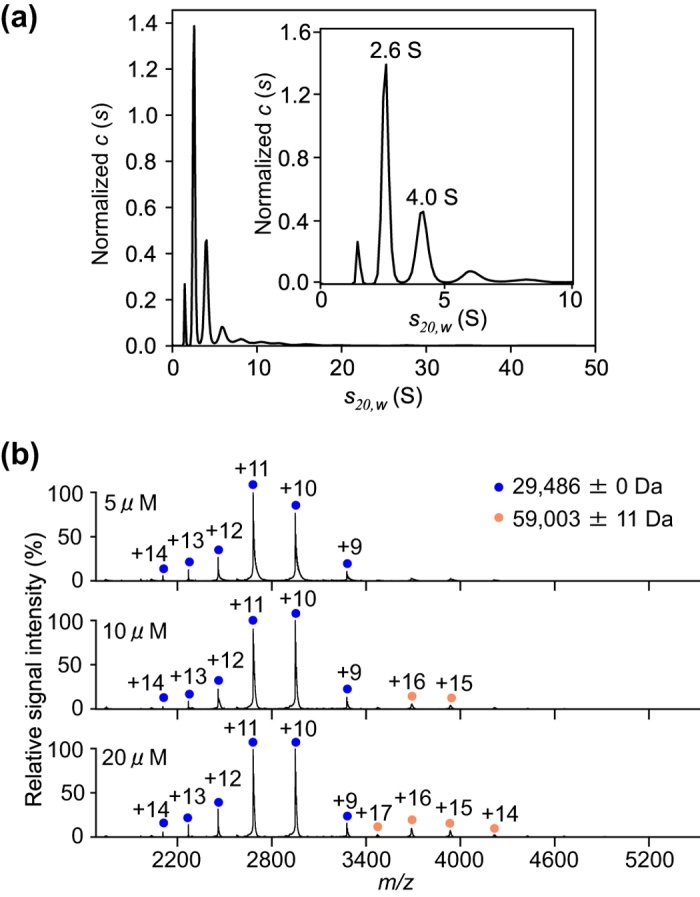
Characterization of the oligomeric state of the α6 subunit. (**a**) Distribution of α6 sedimentation coefficients derived from SV-AUC experiments. Inset shows the enlarged view with *s*-ranging from 0–10 displaying the peaks corresponding to monomer (2.6 S) and dimer (4.0 S) of α6. (**b**) Mass spectra of α6 at 5, 10, and 20 μM under non-denaturing conditions. Blue and orange circles show the ion series of the α6 monomer and dimer, respectively.

Furthermore, we examined the oligomeric state of the human proteasome α6 subunit, which makes direct contact with the α7 subunit in the proteasomal hetero-heptameric α-ring. The SV-AUC analysis showed that a majority of α6 exists as a monomer (2.6 S peak), while indicating that this subunit has a tendency to aggregate (Fig. 3a). The mass spectrum of α6 under non-denaturing conditions exhibited one major and one minor ion series, corresponding to the molecular masses of the monomer (29,486 ± 0 Da) and dimer (59,003 ± 11 Da), respectively (Fig. 3b).

To address the structural basis of the distinct oligomeric properties between α6 and α7, we made a hypothetical model of two α6 subunits brought into juxtaposition based on the homo-tetradecameric structure of α7 (Supplementary Fig. S1). Although a detailed comparison of the inter-subunit interaction modes was difficult because of the low resolution of the present crystal structure, the model clearly shows that α6 makes steric hindrances at the inter-subunit interface, thereby explaining why α6 is not able to form the homo-heptameric ring as α7 does.

### Disassembly of the α7 double ring upon the addition of α6

**Figure 4:**
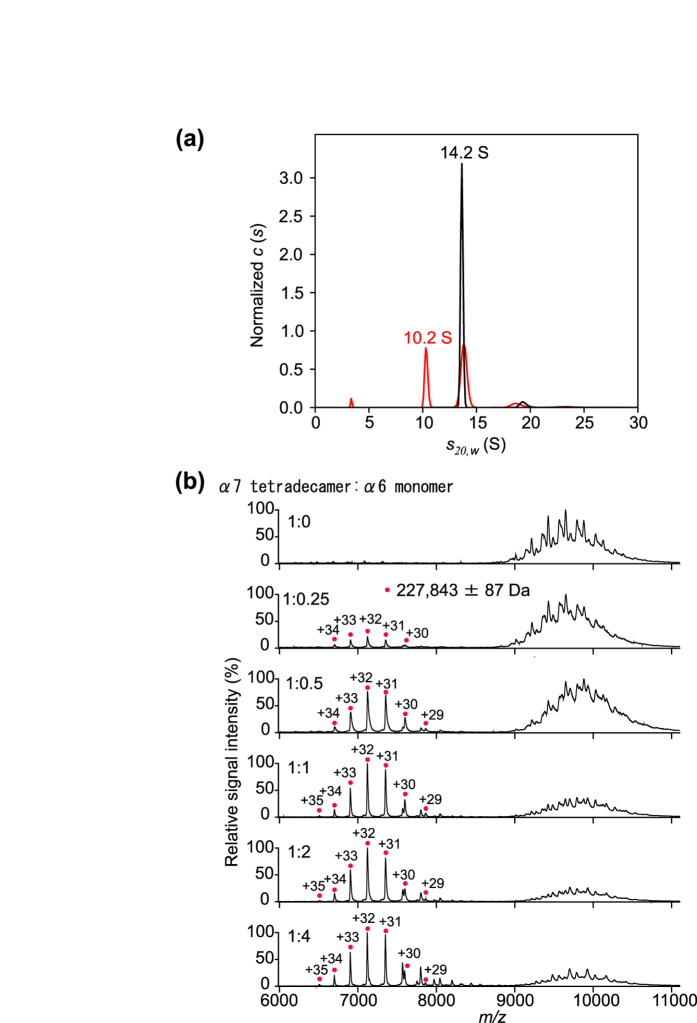
Characterization of the complex of α7 and α6 subunits. (**a**) Distribution of sedimentation coefficients of mixtures of α7 and α6 at 1:0 (black line) and 1:4 (red line) molar ratios (α7 tetradecamer to α6 monomer) derived from SV-AUC experiments. The 10.2 S and 14.2 S peaks correspond to the 1:7 hetero-octameric complexes of α6 and α7 and the α7 homo-tetradecamer, respectively. (**b**) Mass spectra of mixtures of α7 and α6 at 1:0, 1:0.25, 1:0.5, 1:1, 1:2, and 1:4 molar ratios (α7 tetradecamer to α6 monomer). Magenta circles show the ion series of the 1:7 hetero-octamer complexes of α6 and α7.

Further, we attempted to characterize the oligomeric states of a mixture of α7 and α6 using SV-AUC and MS. The SV-AUC data indicated that the α7 homo-tetradecamer was less populated in the presence of α6, with the appearance of a smaller complex with a sedimentation coefficient of 10.2 S (Fig. 4a). In the MS analysis, the ion peak intensities of the α7 homo-tetradecamer were attenuated on titration with α6, with concomitant appearance of a new peak series (Fig. 4b). The molecular mass determined for this complex was 227,843 ± 87 Da, which corresponds to a 7:1 complex of α7 and α6 (with a calculated mass of 227,394 Da). All these data indicate that α6 interacts with α7, disassembling one mole of the tetradecameric double-ring of α7 and thereby give rise to two moles of the 1:7 hetero-octameric α6–α7 complex. The mass spectrometric data showed that the α7 homo-tetradecamer was residual even in the presence of excess amount of α6, indicating that α7 was under equilibrium between the homo-tetradecameric state and the hetero-octameric form with α6 under the experimental condition. The mass spectra acquired at 60 min (upper) and 115 min (lower) after mixing of α6 and α7 were virtually identical in terms of the population of the α7 homo-tetradecamer and the α6-α7 hetero-octamer, implying that the system reached equilibrium within 60 min (Supplementary Fig. S2). The sedimentation coefficient of a single homo-heptameric α7 ring was calculated to be 8.9 S from the present crystal structure, suggesting that the 10.2 S hetero-octamer had a ring-shaped structure, although the position of α6 was uncertain.

Two alternative models for the disassembly of homo-heptameric α7 could be hypothesized. In one model, α7 exists in equilibrium between the double- and single-ring forms and α6 traps and stabilizes the single-ring species. However, our previous SANS data indicated that no detectable ring exchange occurred for 14 h after mixing deuterated and non-deuterated α7 homo-tetradecamers, indicating that the two heptameric rings are tightly associated with each other in the absence of the α6 subunit^15^. The other model is that α6 binds the α7 homo-tetradecamer and induces some type of allosteric conformational change at the ring–ring interface, resulting in the disassembly of the double-ring structure. Considering the stability of the α7 double-ring form in the absence of α6, the latter model is more plausible, although an α6-bound homo-tetradecameric species of α7 could be detected in neither the SV-AUC profiles nor the mass spectra. Further kinetic and structural studies are necessary to identify the disassembly mechanism and quaternary structure of the α6–α7 hetero-octamer.

In summary, the present study demonstrates that the proteasome α6 subunit acts as a breaker of the α7 double-ring structure. Our findings suggest that proteasome formation involves disassembly processes of non-native oligomeric forms of proteasome subunits as assembly intermediates. The proteasome assembly pathway is a potential target for anticancer drug development^16^,^17^,^18^. Therefore, our findings will provide new clues for drug discovery targeting the assembly/disassembly intermediates generated during proteasome formation.

## Methods

### Protein expression and purification

Human proteasome α6 short isoform and α7 subunits were expressed using *Escherichia coli* strain BL21-CodonPlus and purified as described previously^10^,^15^. For the preparation of recombinant proteins, the cells were grown in Luria–Bertani medium. After sonication and centrifugation, cell lysates were subjected to anion-exchange chromatography (DEAE Sepharose Fast Flow, GE Healthcare). The proteins were further purified using an anion-exchange HPLC column (RESOURCE Q, GE Healthcare) and then with a gel-filtration HPLC column (HiLoad 26/60 Superdex 200 pg, GE Healthcare).

### Crystallization, X-ray data collection, and structure determination

Native and selenomethione (SeMet)-substituted human α7 (10 mg/mL) was dissolved in 50 mM Tris-HCl (pH 7.4) and 150 mM NaCl. Crystals were grown in a buffer containing 24% PEG400, 100 mM Tris-HCl (pH 7.5), and 0.2 M magnesium chloride. These mixtures were incubated at 20 °C for approximately 1 week. The crystals were cryoprotected with the reservoir solution and flash-cooled in liquid nitrogen. The native and SeMet-substituted crystals belonged to space group *P*4_3_2_1_2, with one α7 tetradecamer per asymmetric unit. They diffracted at resolutions up to 3.75- and 4.20-Å resolution, respectively. Diffraction data were scaled and integrated using HKL2000^19^.

The 3.75-Å resolution crystal structure of the α7 tetradecamer was solved by the molecular replacement method using the program MOLREP^20^ with a single α-ring of archaeal proteasome (Protein Data Bank code 1J2P)^14^ as the search model. Subsequently, fourteen bovine α7 subunit copies (PDB code 1IRU)^5^ were superimposed and then replaced with the archaeal α subunits. Model fitting to the electron density maps was performed using COOT^21^, in conjunction with the SeMet anomalous data. REFMAC5^22^ was used for the crystal structure refinement, and the stereochemical quality of the final model was validated using PROCHECK^23^. The crystal parameters and refinement statistics are summarized in Supplementary Table S1. The molecular graphics were prepared using PyMOL (http://www.pymol.org/).

### Sedimentation velocity analytical ultracentrifugation

Sedimentation analytical ultracentrifugation experiments were performed in 150 mM potassium phosphate (pH 7.4), using a ProteomeLab XL-I Analytical Ultracentrifuge equipped with four-hole An60 Ti rotor (Beckman Coulter). Solutions loaded in epon or aluminum centerpieces (Beckman Coulter) were run at 25,000 rpm for α7 and α7 + α6, and at 55,000 rpm for α6. Data were collected using an absorbance optical system at wavelengths where absorbance values were between 0.8 and 1.2. Data were analyzed using the continuous c (*s*) distribution model in the program SEDFIT (version 14.4d). The partial specific volume of α6 and α7, buffer viscosity, and buffer density, calculated using the program SEDNTERP 1.09, were 0.73035 cm^3^/g, 0.71588 cm^3^/g, 1.013 cP, and 1.0009 g/mL, respectively. Hydrodynamic parameters were calculated from the crystallographic data with a program SOMO (SOlution MOdeller) equipped with the UltraScan 3^12^.

### Mass spectrometry under non-denaturing conditions

The purified α7 and α6 proteins (420 μM and 510 μM monomer, respectively) were buffer-exchanged into 10 mM ammonium acetate, pH 6.8, and 150 mM ammonium acetate, pH 7.5, respectively, by passing the proteins through a Bio-Spin 6 column (Bio-Rad). The buffer-exchanged α7 (28 μM monomer) and α6 (5, 10, and 20 μM monomer) proteins were immediately analyzed by nanoflow electrospray ionization MS using gold-coated glass capillaries made in house (approximately 2–5 μL sample loaded per analysis). Buffer-exchanged α7 (2 μM tetradecamer) and α6 (0.5, 1, 2, 4, and 8 μM monomer) at pH 7.5 were mixed, incubated at 20 °C for 1 h, and analyzed by nanoflow electrospray ionization MS. Spectra were recorded on a SYNAPT G2-S*i* HDMS mass spectrometer (Waters, Manchester, UK) in positive ionization mode at 1.33 kV with a 150 V sampling cone voltage and source offset voltage, 0 V trap and transfer collision energy, and 5 mL/min trap gas flow. The spectra were calibrated using 1 mg/mL cesium iodide and analyzed using Mass Lynx software (Waters).

## Additional Information

**Accession codes:** The coordinate and structural factor of the crystal structure of human α7 homo-tetradecamer has been deposited in the Protein Data Bank under accession number 5DSV.

**How to cite this article**: Ishii, K. *et al.* Disassembly of the self-assembled, double-ring structure of proteasome α7 homo-tetradecamer by a6. *Sci. Rep.* **5**, 18167; doi: 10.1038/srep18167 (2015).

## Supplementary Material

Supplementary Information
