# Supplementary material for: Disassembly of the self-assembled, double-ring structure of proteasome α7 homo-tetradecamer by α6
Source: Sci Rep. 2015 Dec 14;5:18167. doi: 10.1038/srep18167 (PMC4677347; doi:10.1038/srep18167)
Supplement: Supplementary Information [file srep18167-s1.pdf]

Supplementary information

Disassembly of the self-assembled, double-ring structure of proteasome α7 homo-tetradecamer by α6

Kentaro Ishii[1], Masanori Noda[2], Hirokazu Yagi[3], Ratsupa Thammaporn[4,5], Supaporn Seetaha[4,5], Tadashi Satoh[3,6], Koichi Kato[1,3,5,*], and Susumu Uchiyama[1,2,*]

[1]Okazaki Institute for Integrative Bioscience, National Institutes of Natural Sciences, 5-1 Higashiyama, Myodaiji, Okazaki, Aichi 444-8787, Japan, [2]Department of Biotechnology, Graduate School of Engineering, Osaka University, 2-1 Yamadaoka, Suita, Osaka 565-0871, Japan, [3]Graduate School of Pharmaceutical Sciences, Nagoya City University, 3-1 Tanabe-dori, Mizuho-ku, Nagoya, Aichi 467-8603, Japan, [4]Faculty of Science, Kasetsart University, Bangkean, Bangkok 10900, Thailand, [5]Institute for Molecular Science, National Institutes of Natural Sciences, 5-1 Higashiyama, Myodaiji, Okazaki, Aichi 444-8787, Japan, [6]JST, PRESTO, 3-1 Tanabe-dori, Mizuho-ku, Nagoya, Aichi 467-8603, Japan.

Table S1. Data collection and refinement statistics for human proteasome α7 homo-tetradecamer

	Native	SeMet
**Crystallographic data**		
Space group	$P4_32_12$	$P4_32_12$
Unit cell *a*/*b*/*c* (Å)	132.5/132.5/444.6	133.3/133.3/443.7
α/β/γ (°)	90.0/90.0/90.0	90.0/90.0/90.0
**Data processing statistics**		
Beam line	NSRRC 13B1	SPring-8 BL44XU
Wavelength (Å)	1.00000	0.97873
Resolution (Å)	50-3.75 (3.81-3.75)	50-4.20 (4.27-4.20)
Total/unique reflections	198,252/41,746	844,780/30,234
Completeness (%)	97.1 (98.5)	99.9 (100)
R_{merge} (%)	8.1 (49.7)	11.1 (45.1)
I / σ (I)	16.3 (2.5)	75.7 (14.9)
Redundancy	4.9 (5.0)	28.0 (29.4)
**Refinement statistics**		
Resolution (Å)	20.0-3.75	
R_{work} / R_{free} (%)	21.0/29.1	
R.m.s. deviations from ideal		
Bond lengths (Å)	0.010	
Bond angles (°)	1.23	
Ramachandran plot (%)		
Most favored	88.3	
Additionally allowed	10.9	
Generously allowed	0.8	
Disallowed	0	

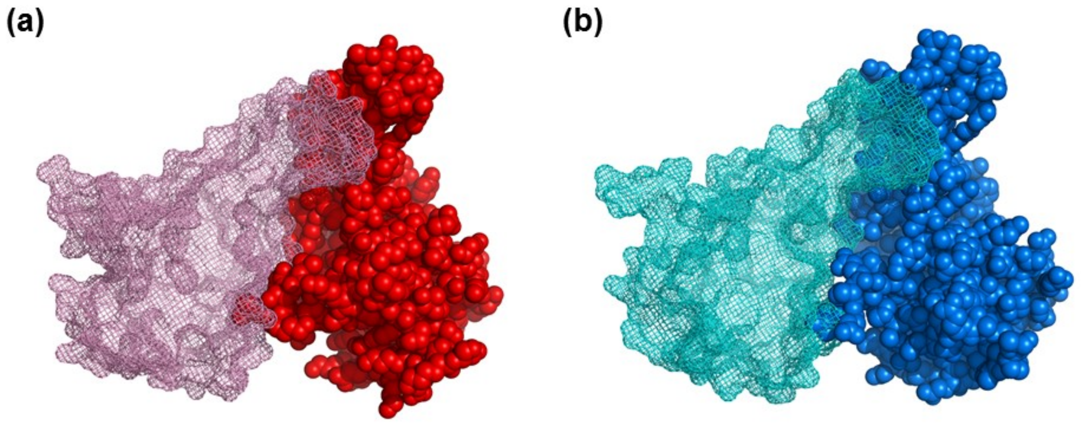

Figure S1. Comparison of α7/α7 and α6/α6 inter-subunit interactions. (a) A model of α7 homo-dimer derived from the crystal structure of the α7 homo-tetradecamer. (b) A model of α6 homo-dimer constructed by superimposition of the α6 subunit derived from the human 20S proteasome (PDB code: 4R3O) to the α7 homo-dimer as a template. In each model, one subunit is represented by sphere, while the other subunit is shown by mesh.

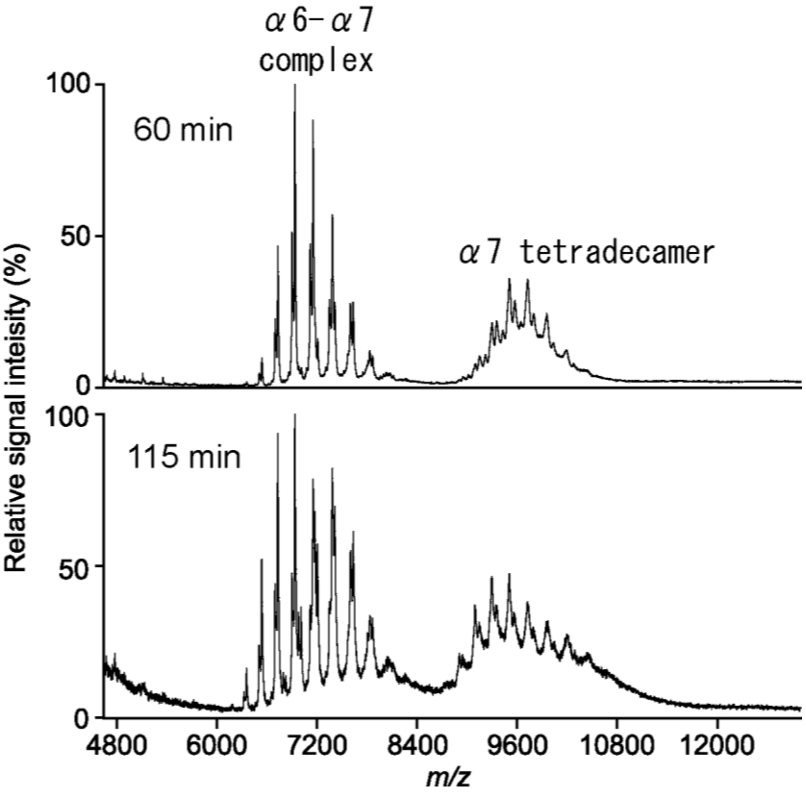

Figure S2. Mass spectra of mixtures of α7 and α6 at 1:4 molar ratios (α7 tetradecamer to α6 monomer) acquired at 60 min (upper) and 115 min (lower) after mixing.
